# Biomechanical Evaluation of Oblique Lumbar Interbody Fusion with Various Fixation Options: A Finite Element Analysis

**DOI:** 10.1111/os.12877

**Published:** 2021-02-22

**Authors:** Chengjie Song, Hengrui Chang, Di Zhang, Yingze Zhang, Mingxin Shi, Xianzhong Meng

**Affiliations:** ^1^ Department of Spinal Surgery The Third Hospital of Hebei Medical University ShiJiazhuang China

**Keywords:** Biomechanical, Cage stress, Endplate stress, Finite element analysis, Oblique lumbar interbody fusion

## Abstract

**Objective:**

The aim of the present study was to clarify the biomechanical properties of oblique lumbar interbody fusion (OLIF) using different fixation methods in normal and osteoporosis spines.

**Methods:**

Normal and osteoporosis intact finite element models of L_1_–S_1_ were established based on CT images of a healthy male volunteer. Group A was the normal models and group B was the osteoporosis model. Each group included four subgroups: (i) intact; (ii) stand‐alone cage (Cage); (iii) cage with lateral plate and two lateral screws (LP); and (iv) cage with bilateral pedicle screws and rods (BPSR). The L_3_–L_4_ level was defined as the surgical segment. After validating the normal intact model, compressive load of 400 N and torsional moment of 10 Nm were applied to the superior surface of L_2_ to simulate flexion, extension, left bending, right bending, left rotation, and right rotation motions. Surgical segmental range of motion (ROM), cage stress, endplate stress, supplemental fixation stress, and stress distribution were analyzed in each group.

**Results:**

Cage provided the minimal reduction of ROM among all motions (normal, 82.30%–98.81%; osteoporosis, 92.04%–97.29% of intact model). BPSR demonstrated the maximum reduction of ROM (normal, 43.94%–61.13%; osteoporosis, 45.61%–62.27% of intact model). The ROM of LP was between that of Cage and BPSR (normal, 63.25%–79.72%; osteoporosis, 70%–87.15% of intact model). Cage had the minimal cage stress and endplate stress. With the help of LP and BPSR fixation, cage stress and endplate stress were significantly reduced in all motions, both in normal and osteoporosis finite element models. However, BPSR had more advantages. For cage stress, BPSR was at least 75.73% less than that of Cage in the normal model, and it was at least 80.10% less than that of Cage in the osteoporosis model. For endplate stress, BPSR was at least 75.98% less than that of Cage in the normal model, and it was at least 78.06% less than that of Cage in the osteoporosis model. For supplemental fixation stress, BPSR and LP were much less than the yield strength in all motions in the two groups. In addition, the comparison between the two groups showed that the ROM, cage stress, endplate stress, and supplemental fixation stress in the normal model were less than in the osteoporosis model when using the same fixation option of OLIF.

**Conclusion:**

Oblique lumbar interbody fusion with BPSR provided the best biomechanical stability both in normal and osteoporosis spines. The biomechanical properties of the normal spine were better than those of the osteoporosis spine when using the same fixation option of OLIF.

## Graphical abstract

In this study, we established OLIF finite element models with various fixation options in normal and osteoporosis spines, and found that OLIF with BPSR fixation had the best biomechanical properties.

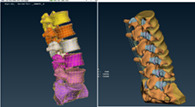

## Introduction

Although lumbar fusion has been used in clinic decades of history, both anterior and posterior approaches have disadvantages. Posterior surgery can easily damage nerve roots and destroy the normal structure, leading to long‐term pain and discomfort in the waist^1^, ^2^, ^3^, ^4^. The procedures of anterior surgery are complex and the incidence of complications is high. During surgery, the large blood vessels in the abdomen can be damaged, which causes hemorrhaging and can even be life‐threatening; if the vas deferens is damaged, retrograde ejaculation may occur^5^, ^6^, ^7^. Using the recently developed oblique lumbar interbody fusion (OLIF) technique, the target segment can be reached from the retroperitoneal space through the front of the psoas major muscle, avoiding direct damage to the spinal canal and nerve, with minimally invasive characteristics, such as small incisions, less bleeding, and short hospital stay^8^, ^9^, ^10^, ^11^.

Oblique lumbar interbody fusion was introduced by Silvestre in 2012^12^; it not only has advantages in surgical approach but also enables the implantation of a larger cage, increases the intervertebral fusion rate, and reduces the risk of subsidence of the cage. Since its introduction, it has been welcomed by spinal physicians and widely used in the treatment of degenerative diseases of the lumbar spine^13^, ^14^, ^15^. Although OLIF is popular, there are few studies on the biomechanical stability of various fixation options of OLIF, and it is not clear which fixation option has the best biomechanical properties. In addition, there are increasing numbers of patients with osteoporosis of the spine, with a rising trend in younger age groups. However, the biomechanical stability provided by different fixation methods of OLIF for osteoporosis has not been reported.

At present, there are two research methods used for spine biomechanics: studies of *in vitro* specimens and finite element studies. Studying *in vitro* specimens is a basic biomechanics research method. However, experiments take a long time because of the difficulty of obtaining specimens. Moreover, tissues such as muscles and ligaments are prone to fatigue, and the method has poor repeatability^16^, ^17^, ^18^. Many scholars have begun since the 1990s to use the finite element method to study biomechanics. The finite element method can establish different surgical models using computer software and simulate the transient postoperative state by imposing different boundaries and loading conditions. Not only can the experimental conditions be artificially controlled, but the operation can be repeated. A disadvantage of the finite element model (FEM) is that it disregards the roles of soft tissue. However, the experimental results obtained by the validated FEM are still of certain significance. Brekelmans *et al*.^19^ first applied the finite element techniques in the field of orthopaedics. Liu *et al*.^20^ took the lead in establishing a three‐dimensional FEM of the lumbar spine. With the development of computer technology, the accuracy of FEM is improving, and its contribution has been recognized by numerous scholars. Considering the repeatability and operability of the finite element method, we chose to use it to research the biomechanical properties of OLIF with different fixation methods.

In this study, the three‐dimensional finite element method was used to establish the following fixation models: OLIF stand‐alone cage (Cage), cage with lateral plate and two lateral screws (LP), and cage with bilateral pedicle screws and rods (BPSR) for normal and osteoporosis spines. By comparing the surgery segmental ROM, cage stress, endplate stress, supplemental fixation stress, and stress distribution, we aimed to: (i) determine which fixation method in the normal model had the best biomechanical performance; (ii) determine which fixation method in the osteoporosis model has the best biomechanical performance; and (iii) compare the difference in biomechanical properties of normal and osteoporotic models when using the same fixation method.

## Methods

### *Construction of an Intact Lumbar Finite Element Model*

In this study, a 28‐year‐old healthy male volunteer (weight 65 kg, height 173 cm, and without lumbar disease) underwent CT scanning, with slice thickness of 0.625 mm. A total of 570 CT images were processed using commercial software (Mimics 20.0; Materialize, Leuven, Belgium) create a solid model. After repair, Hypermesh (Altair Technologies, Fremont, CA, USA) was used to mesh the solid model of the bony and ligamentous structures. Finally, Abaqus (Simulia., Providence, RI, USA) was used for material property definitions, model assembly and FEM analysis.

**Fig. 1:**
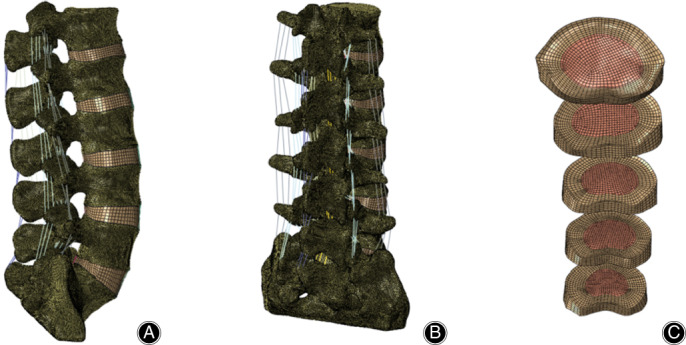
Normal finite element model of L_1_–S_1_. (A) Side view. (B) Dorsal view. (C) Intervertebral disc.

The FEM (Fig. 1 **)** included L_1_–S_1_ vertebrae, intervertebral discs and the ligaments system. The vertebral body included cortical bone, cancellous bone, and posterior bone. The thickness of the cortical bone was 1 mm^21^. The intervertebral discs were separated into annulus fibrosus, nucleus pulposus, and superior and inferior endplates. The discs were defined to be composed of 44% nucleus pulposus and 56% annulus fibrosus based on histological data^22^, and the thickness of the endplate was 0.5 mm^23^. The ligaments included the anterior longitudinal ligament, the posterior longitudinal ligament, the ligamentum flavum, the interspinous ligament, the supraspinal ligament, the capsular ligaments, and the intertransverse ligament. They were set as truss elements (T3D2) subjected only to tensile load. The FEM was meshed using the tetrahedral and hexahedral elements, except for the ligaments. There were 1,352,850 elements and 268,908 nodes. The material properties of components are shown in Table 1 ^22^, ^24^, ^25^, ^26^, ^27^. For “osteoporosis spine”, the elastic modulus of cortical bone and cancellous bone decreased by 33% and 66%, respectively, compared with the normal intact model^11^. Group A was the normal model and group B was the osteoporosis model.

**TABLE 1:** Material properties assigned to the FEM

Components	Young's modulus (Mpa)	Poisson ratio
Cortical bone	12,000	0.3
Cancellous bone	100	0.2
Endplate	4000	0.3
Nucleus pulposus	1	0.49
Annulus	4.2	0.45
Anterior longitudinal ligament	20	0.3
Posterior longitudinal ligament	20	0.3
Ligamentum flavum	19.5	0.3
Interspinous ligament	11.6	0.3
Supraspinous ligament	15	0.3
Transverse ligament	58.7	0.3
Capsular ligament	32.9	0.3
Cage (polyetheretherketone)	3500	0.3
Pedicle screws and rod	110000	0.3
Lateral plate and screws	110000	0.3

FEM, finite element model.

### *Construction of Surgical Finite Element Model*

**Fig. 2:**
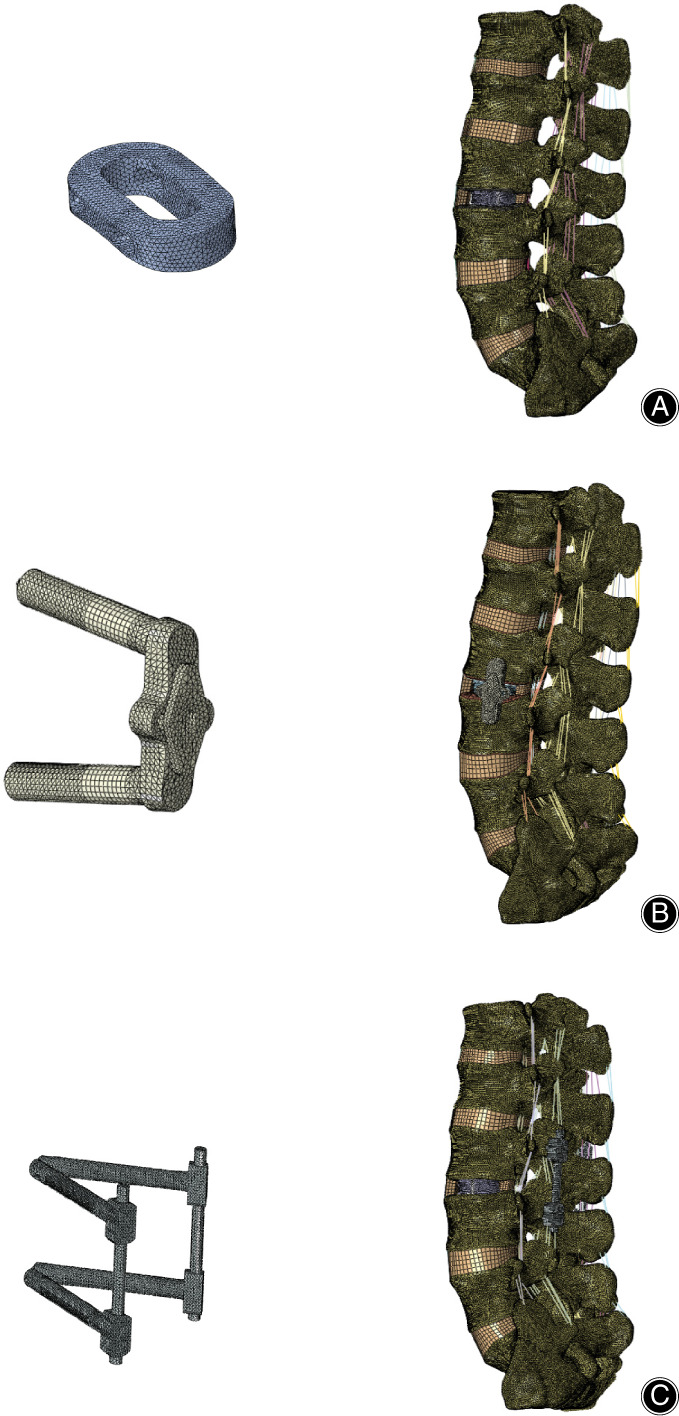
Finite element model with various fixation options. (A) Cage, stand‐alone cage. (B) LP, cage with lateral plate and two lateral screws. (C) BPSR, cage with bilateral pedicle screws and rods.

The L_3_–L_4_ level was defined as the surgical segment, and the annulus fibrosus, the nucleus pulposus, and the cartilage endplate were removed from the left side. Then, the supplemental fixation models of stand‐alone cage (Cage), cage with lateral plate and two lateral screws (LP), and cage with bilateral pedicle screws and rods (BPSR) were constructed, respectively, for the normal and osteoporosis intact models. There were eight FEM. The normal FEM with various fixation options are shown in Fig. 2.

The interbody cage and supplemental fixations were tetrahedral mesh. The cage was modeled based on Oracle cage (DePuy Synthes). It had 8° lordosis, was 40 mm long, 22 mm wide, 11 mm high anteriorly and 8 mm posteriorly, and it was made of polyetheretherketone. The lateral plate and two lateral screws were modeled based on a double‐medical plate, and the diameter of the lateral screws was 6.5 mm and 35 mm long. The bilateral pedicle screws and rods were modeled based on the EXPEDIUM 5.5 System (DePuy Synthes). The diameter of the pedicle screw was 5.5 mm and the length was 50 mm. The diameter of the rod was 5.5 mm, and the length exceeded the distance between the upper and lower pedicle screws. The lateral plate, screws, and rods were made of titanium alloy (Ti6Al4V). The material properties of the implant components are listed in Table 1.

### *Boundary and Loading Conditions*

All the surgical models assumed that there was no sliding between the cage and the contact surfaces of the upper and lower vertebral bodies, and frictional contact was set between the articular process and the structure, and between the screws and the screw holes. The friction coefficient was set as 0.2. The lower surface of the S_1_ vertebral body was fixed. So that the finite element research was closer to reality, an axial compressive preload of 400 N was added to the superior surface of L_2_ to simulate the physiological compression, and a torsional moment of 10 Nm was imposed to simulate the motion of flexion, extension, left bending (LB), right bending (RB), left rotation (LR), and right rotation (RR). The ROM of the L_3_–L_4_ segment was recorded and compared with the intact . Cage stress, endplate stress, supplemental fixation stress, and the stress distribution were compared in different surgical modes.

### *Finite Element Model validation*

The L_3_–L_4_ segment ROM for different motions of the normal intact model was compared with previous outcomes of Yamamoto^28^ to verify the validity of the model. It was determined that the L_3_–L_4_ ROM of the normal intact model was similar to that in the previous study, verifying that the normal intact FEM was valid.

## Results

### *Range of motion*

#### *Normal Model*

For all surgical constructs, the L_3_–L_4_ ROM was decreased compared with the intact in all motions. Cage provided the minimal reduction of ROM, which was 83.81% in flexion, 98.81% in extension, 84.07% in LB, 87.98% in RB, 88.55% in LR, and 91.24% in RR of the intact spine. The ROM of LP was slightly less than that of Cage. It was 79.73% in flexion, 74.55% in extension, 64.58% in LB, 66.13% in RB, 63.25% in LR, and 67.37% in RR of the intact spine. BPSR showed the maximum reduction of ROM compared with the intact; it was 50.38% in flexion, 43.94% in extension, 54.24% in LB, 51.90% in RB, 61.14% in LR, and 59.52% in RR of the intact spine.

#### *Osteoporosis Model*

For osteoporosis FEM, the ROM of Cage was 94.88%, 94.83%, 92.94%, 92.04%, 93.40%, and 97.30% of the intact spine in flexion, extension, LB, RB, LR, and RR, respectively. The LP ROM was less than that of Cage, greater than that of BPSR, and was 86.59% in flexion, 87.15% in extension, 70.00% in LB, 78.38% in RB, 72.82% in LR, and 78.10% in RR of the intact spine. BPSR also provided the maximum reduction of ROM compared with the intact for all motions, and it showed 45.61%, 45.81%, 59.41%, 51.05%, 62.27%, and 62.16% of the intact spine in flexion, extension, LB, RB, LR, and RR, respectively. Table 2 describes the L_3_–L_4_ segment ROM for the normal and osteoporosis models.

**TABLE 2:** L_3_–_4_ segment ROM (°) of the normal and osteoporosis models

	Normal				Osteoporosis			
	Intact	Cage	LP	BPSR	Intact	Cage	LP	BPSR
Flexion	6.61	5.44	5.27	3.33	8.20	7.78	7.10	3.74
Extension	5.03	4.97	3.75	2.21	7.16	6.79	6.24	3.28
LB	5.90	4.96	3.81	3.20	6.80	6.32	4.76	4.04
RB	4.99	4.39	3.30	2.59	6.66	6.13	5.22	3.40
LR	3.32	2.94	2.10	2.03	3.79	3.54	2.76	2.36
RR	3.31	3.02	2.23	1.97	3.70	3.60	2.89	2.30

BPSR, cage with bilateral pedicle screws and rods; Cage, stand‐alone cage; LB, left bending; LP, cage with lateral plate and two lateral screws; LR, left rotation; RB, right bending; ROM, range of motion; RR, right rotation.

### *Cage Stress*

#### *Normal Model*

**Fig. 3:**
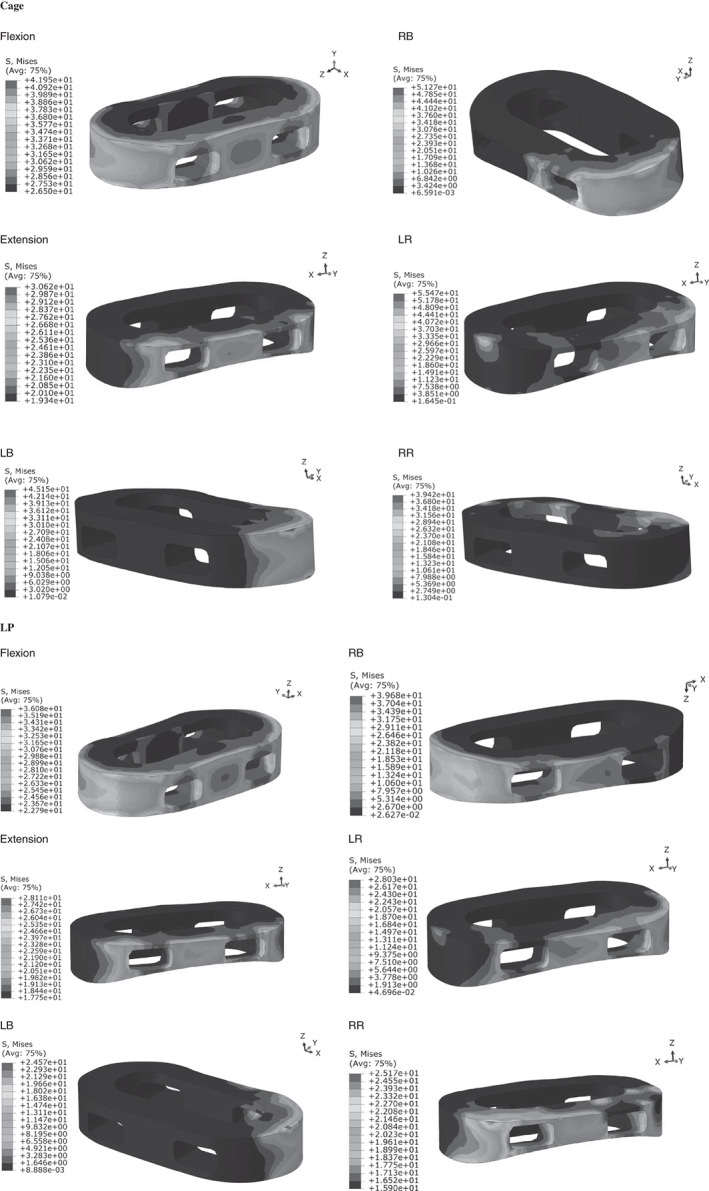
(A) Normal model cage stress distribution for six motions. (B) Osteoporosis model cage stress distribution for six motions. BPSR, cage with bilateral pedicle screws and rods; Cage, stand‐alone cage; LB, left bending; LP, cage with lateral plate and two lateral screws; LR, left rotation; RB, right bending; RR, right rotation.

For normal FEM, Cage provided the greatest cage stress and BPSR provided minimal cage stress; the cage stress of LP was slightly less than that of Cage, except for flexion and extension, in the normal model. The cage stress of BPSR was 71.66% less than that of Cage in flexion, 75.73% in extension, 64.61% in LB, 68.36% in RB, 64.65% in LR, and 60.36% in RR. The cage stress distribution is displayed in Fig. 3A; stress was distributed in the periphery of cage for all motions.

#### *Osteoporosis Model*

The cage stress trends of the osteoporosis FEM were consistent with those of the normal model. The cage stress of BPSR was 65.91%, 80.10%, 59.71%, 52.24%, 71.36%, and 65.29% less than that of Cage in flexion, extension, LB, RB, LR, and RR, respectively. The cage stress distribution is shown in Fig. 3B; it was distributed in the periphery of cage. Table 3 describes the cage stress of normal and osteoporosis models.

**TABLE 3:** Cage stress (Mpa) for different fixation options of the normal and osteoporosis models

	Normal			Osteoporosis		
	Cage	LP	BPSR	Cage	LP	BPSR
Flexion	41.95	36.08	11.89	43.30	39.65	14.76
Extension	30.62	28.11	7.43	32.81	30.08	6.53
LB	45.15	24.57	15.98	53.43	35.53	15.30
RB	51.27	39.68	16.22	46.10	38.45	16.00
LR	55.47	28.03	19.61	53.12	36.19	21.40
RR	39.42	25.17	18.04	38.17	30.54	18.23

BPSR, cage with bilateral pedicle screws and rods; Cage, stand‐alone cage; LB, left bending; LR, left rotation; RB, right bending; RR, right rotation.

### *Endplate Stress*

Cage subsidence often occurs in the superior endplate after the operation, so we recorded the stress of the L_4_ superior endplate (Table 4).

**TABLE 4:** Endplate stress (Mpa) different fixation options of the normal and osteoporosis models

	Normal			Osteoporosis		
	Cage	LP	BPSR	Cage	LP	BPSR
Flexion	37.34	34.40	9.79	41.93	37.37	15.57
Extension	27.27	25.17	6.55	32.36	31.45	7.10
LB	43.94	21.28	14.56	53.94	33.24	18.30
RB	42.86	29.78	13.48	52.32	39.67	17.10
LR	36.00	16.44	10.35	41.86	25.48	13.20
RR	26.76	17.73	8.60	32.66	20.99	9.10

BPSR, cage with bilateral pedicle screws and rods; Cage, stand‐alone cage; LB, left bending; LP, cage with lateral plate and two lateral screws; LR, left rotation; RB, right bending; RR, right rotation.

#### *Normal Model*

**Fig. 4:**
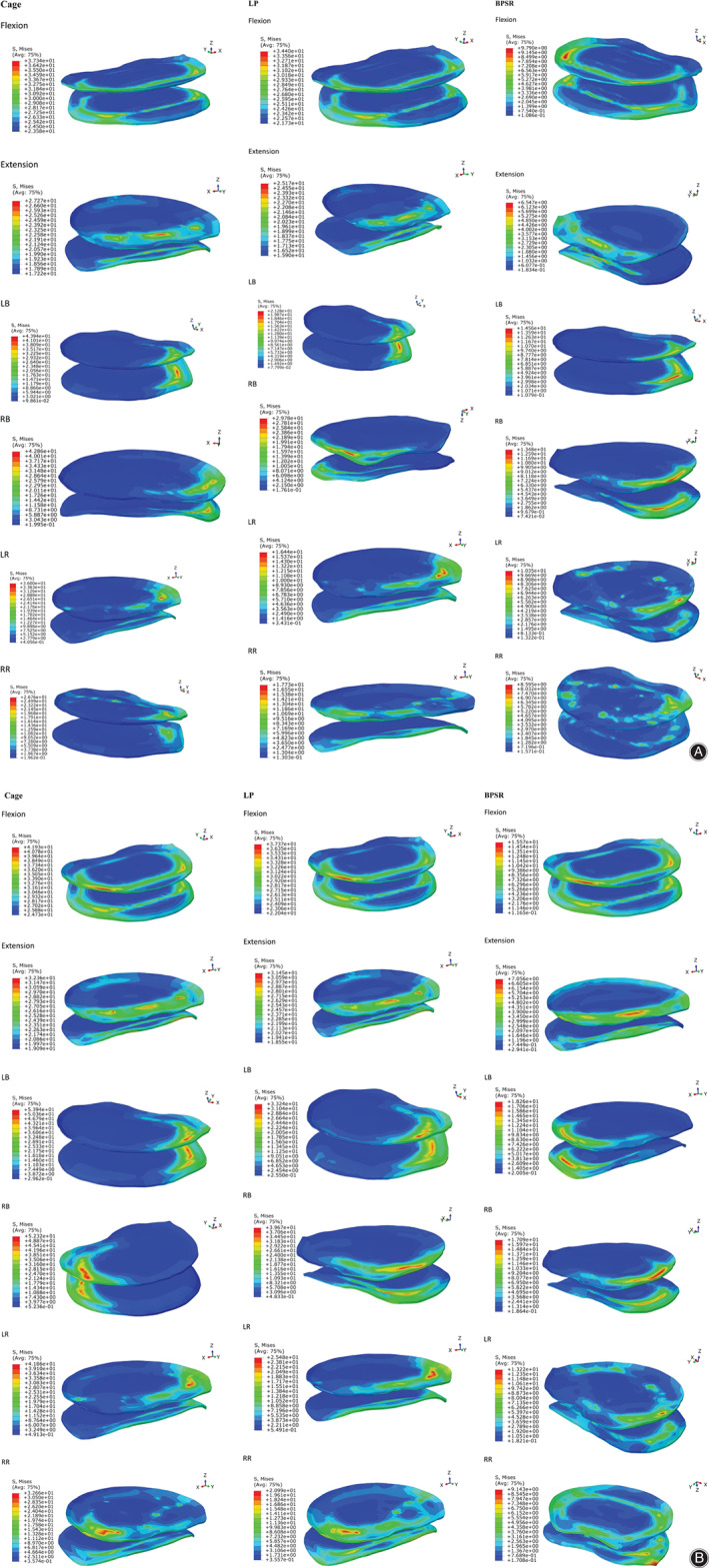
(A) Normal model endplate stress distribution for six motions. (B) Osteoporosis model endplate stress distribution in six motions. BPSR, cage with bilateral pedicle screws and rods; Cage, stand‐alone cage; LB, left bending; LP, cage with lateral plate and two lateral screws; LR, left rotation; RB, right bending; RR, right rotation.

In the three fixation option models, Cage had the greatest endplate stress and BPSR had minimal endplate stress in all motions. Endplate stress of LP was slightly less than that of Cage, except for flexion and extension. The endplate stress of BPSR was 73.78%, 75.98%, 71.25%, 67.86%, 66.86%, and 68.55% less than that of Cage in flexion, extension, LB, RB, LR, and RR, respectively. Figure 4A depicts the endplate stress distribution. Stress was distributed in the endplate periphery for all motions.

#### *Osteoporosis Model*

For the osteoporosis FEM, the stress trends of the fixation methods were the same as for the normal model. That is, Cage had the greatest endplate stress and BPSR had minimal endplate stress in all motions. BPSR endplate stress was 62.87% less than that of Cage in flexion, 78.06% in extension, 68.47% in LB, 72.14% in RB, 66.07% in LR, and 67.32% in RR. Figure 4B shows the endplate stress distribution; it was also distributed in the endplate periphery.

### *Supplemental Fixation Stress*

The supplemental fixation stress included LP stress and BPSR stress (Table 5).

**TABLE 5:** Supplemental fixation stress (Mpa) of normal and osteoporosis model

	Normal		Osteoporosis	
	LP	BPSR	LP	BPSR
Flexion	33.16	50.36	51.52	57.00
Extension	191.40	184.00	146.50	191.30
LB	94.05	158.60	96.67	179.60
RB	66.51	186.60	100.50	207.90
LR	84.49	153.80	96.47	168.10
RR	142.90	181.50	149.10	200.10

BPSR, cage with bilateral pedicle screws and rods; LB, left bending; LP, cage with lateral plate and two lateral screws; LR, left rotation; RB, right bending; RR, right rotation.

#### *Normal model*

**Fig. 5:**
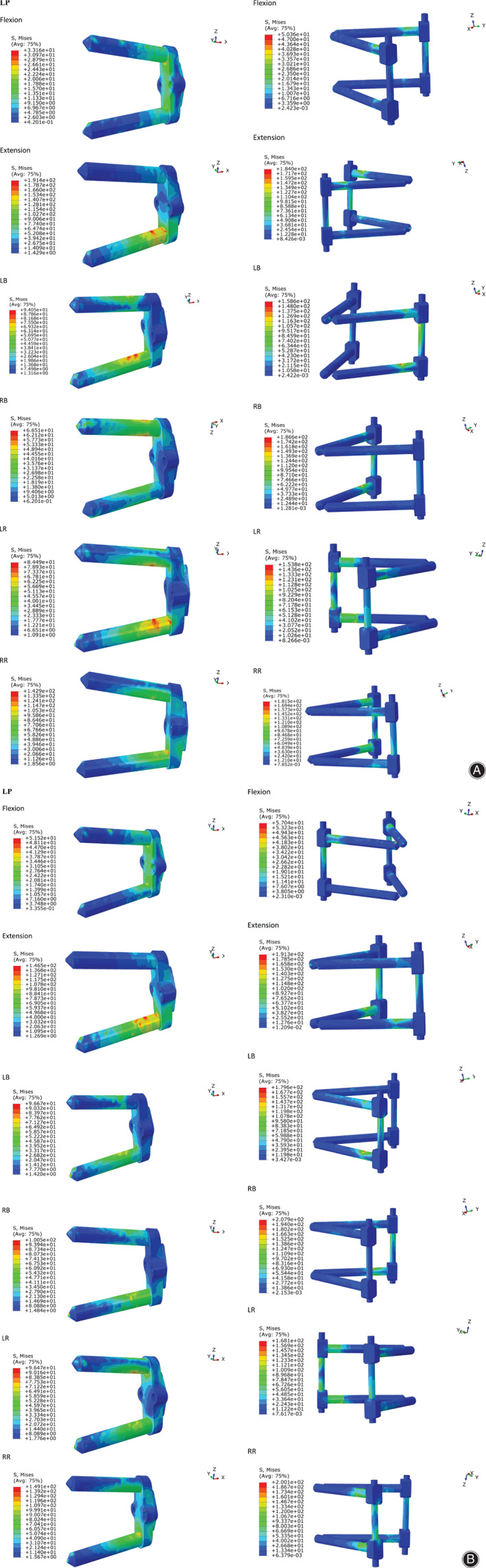
(A) Normal model supplemental fixation stress distribution for six motions. (B) Osteoporotic model supplemental fixation stress distribution for six motions. BPSR, cage with bilateral pedicle screws and rods; Cage, stand‐alone cage; LB, left bending; LP, cage with lateral plate and two lateral screws; LR, left rotation; RB, right bending; RR, right rotation.

For normal FEM, the LP stress was 33.16, 191.4, 94.05, 66.51, 84.49, and 142.9 Mpa in flexion, extension, LB, RB, LR, and RR. The BPSR stress was 50.36 Mpa in flexion, 184.0 Mpa in extension, 158.6 Mpa in LB, 186.6 Mpa in RB, 153.8 Mpa in LR, and 181.5 Mpa in RR. As shown in Fig. 5A, The stress distribution of LP and BPSR were concentrated at the junction of the screw and the vertebra.

#### *Osteoporotic model*

For the osteoporosis models, the stress of LP was 51.52 Mpa in flexion, 146.5 Mpa in extension, 96.67 Mpa in LB, 100.5 Mpa in RB, 96.47 Mpa in LR, and 149.1 Mpa in RR. The BPSR stress was 57.0, 191.3, 179.6, 207.9, 168.1, and 200.1 Mpa in flexion, extension, LB, RB, LR, and RR, respectively. Fig. 5B depicts the LP and BPSR stress distribution of osteoporosis models; it is also concentrated at the junction of the screw and the vertebrae.

## Discussion

Oblique lumbar interbody fusion evolved from lateral lumbar interbody fusion (LLIF)^29^. It has been popular since its introduction due to advantages including preservation of the normal structure of the posterior lumbar, less bleeding, and shorter hospital stay^8^, ^30^. Although OLIF has been successful in the clinic, there are still the risks of cage subsidence, postoperative segment instability, and fracture for patients who undergo posterior supplemental fixation^31^, ^32^. In cases of osteoporosis, these risks may be greater. However, it is not clear which fixing method of OLIF has the best effect. Consequently, we established various fixation options for FEM of normal and osteoporosis spines to determine their biomechanical stability.

Regardless of the approach, the purpose of lumbar fusion is to stabilize the lumbar spine by reducing the ROM of the target segment^33^. In the present study, the results indicate that all the fixation options enhanced the construct stability compared with the intact spine in normal models. Our research showed that in the normal spine group, the degree of stability was greatly different among supplemental fixation models. Cage provided the minimal reduction of ROM in all motions; it was 82.30%–98.81% of the intact model. After the addition of LP, the ROM of the L_3_–L_4_ segment was reduced compared with Cage, but the degrees of reduction of bending and rotation were better than those for flexion–extension. BPSR showed the greatest reduction of ROM in all conditions, and it was 43.94%–61.13% of the intact spine model. The reason for this result is that LP is fixed on the lateral, which has little restriction on the ROM in flexion and extension. BPSR is fixed at the posterior facet joint, which has greater restriction on ROM in various directions. These findings concur with those of with previous LLIF biomechanical studies^34^, ^35^, ^36^. However, in our study, the degree of various fixation options' ROM reduction was smaller compared with previous studies. Possible factors contributing to these differences include individual lumbar differences and different loading conditions. In the osteoporosis spine group, we observed results similar to the those of the normal model. Cage ROM was 92.04%–97.29% of the intact spine, LP ROM was 70%–87.15% of the intact spine, and BPSR ROM was 45.61%–62.27% of the intact spine. Compared with the normal model, no matter what the fixation option, the ROM of the osteoporosis model was larger for all motions. The reason for this difference is that: the elastic modulus of the osteoporosis model was less than that of the normal model; the pullout strength of the internal fixation was weak; and the ROM limitation was not as good as that of the normal model. Therefore, we speculate that the effects of the operation for elderly osteoporosis patients are not as good as for normal patients when the same fixation method is applied.

The greater the stress of the cage and the endplate, the greater the risk of subsidence of the cages into the endplate and the adjoining vertebral bone over time^37^. Malham *et al*. showed that the radiographic subsidence rate of OLIF is approximately 8%^38^. In our study, for a normal spine model, the BPSR option had minimal cage stress and endplate stress. It was indicated that the BPSR risk of cage subsidence was lower compared with LP and Cage. In addition, the cage stress and endplate stress were the greatest after only implanting the stand‐alone cage, indicating that the Cage risk of cage subsidence was the highest compared with LP and BPSR. After lateral plate fixation, the cage stress and endplate stress were obviously decreased compared with the Cage, except for flexion–extension. These findings were comparable to those of previous finite element analysis^33^. However, in the previous finite element study, LP only reduced the cage stress and endplate stress in bending while changing little for other motions. Various types of LP may contribute differently to postoperative stability. Because there is no uniform type of LP at present, we suggest that clinicians select this fixation method with caution. For osteoporosis, we observed similar results to the normal spine model; that is, Cage has the greatest cage stress and endplate stress, and BPSR has the lowest cage stress and endplate stress. Due to the risk of cage subsidence of osteoporosis being higher than normal, the supplemental fixation method with the minimal cage stress and endplate stress should be selected. We suggest the application of OLIF with BPSR to treat osteoporosis. In addition, we observed that with the same fixation method, the cage stress and endplate stress of the osteoporosis model was greater than that of the normal model. Due to the pullout strength of the internal fixation in the osteoporotic model being weak, when external forces exist, the supporting force it provides is small. Therefore, the cage and the endplate are subjected to greater stress. This is also the reason why cage subsidence is more likely to occur in the case of osteoporosis than in a normal spine.

In the present study, in addition to comparing the ROM, the cage stress, and endplate stress, we recorded supplemental fixation stress and stress distribution. Chen *et al*.^39^ showed that the yield strength of titanium alloy was 897–1034 Mpa. Our results showed that bilateral pedicle screws and rod stress and lateral plate and lateral screws stress were far less than the yield strength both in normal and osteoporosis FEM. Implant lateral plates or bilateral pedicle screws are feasible and safe to use. The stress distributions of LP and BPSR were concentrated at the junction of the screw and the vertebrae. To avoid screw breakage or ejection, we suggest that postoperative overactivity be avoided. Moreover, our results show that the supplemental fixation stress in all motions of the osteoporosis model is greater than that of the normal model with the same fixation method, which indicates that the safety of normal patients is better than for osteoporosis patients when using the same fixation option of OLIF.

To our knowledge, this study is the first to apply a three‐dimensional FEM to establish an osteoporotic spine model to research the single‐segment biomechanical stability of OLIF with different fixation methods. Cage and LP provide less stability than BPSR in the osteoporosis FEM, and are comparable to those of the normal FEM. The biomechanical properties of osteoporosis models were not as good as those of normal models when using the same fixation method. Because there are few finite element studies on osteoporosis, our results need to be further confirmed.

This study has some limitations. First, although finite element analysis has many advantages over *in vitro* experiments in the study of spinal biomechanics, the FEM cannot perfectly mimic the human body; for example, the paravertebral soft tissue cannot be precisely recreated and the function of muscles is ignored, which is a common problem faced by all finite element studies. Second, the osteoporosis FEM was constructed by decreasing the elastic modulus of cortical bone and cancellous bone by a certain proportion in normal FEM, ignoring the differences of individuals. Finally, the calculation results of the FEM only reflect the situation directly after the operation. It is not indicative of long‐term postoperative status. Despite some limitations, in this study, the validity of the FEM has been verified, and the different types of fixation have been studied under the same experimental conditions. Therefore, the results of this study are still instructive for clinicians.

### *Conclusion*

Bilateral pedicle screw and rod fixation had advantages in surgical segment ROM, cage stress, and endplate stress compared with Cage and LP fixation for OLIF both in normal and osteoporosis spines, and had the best biomechanical properties. The biomechanical properties of the normal spine were better than those of the osteoporosis spine when using the same fixation method.
